# Antiprogestin mifepristone inhibits the growth of cancer cells of reproductive and non-reproductive origin regardless of progesterone receptor expression

**DOI:** 10.1186/1471-2407-11-207

**Published:** 2011-05-27

**Authors:** Chelsea R Tieszen, Alicia A Goyeneche, BreeAnn N Brandhagen, Casey T Ortbahn, Carlos M Telleria

**Affiliations:** 1 Division of Basic Biomedical Sciences, Sanford School of Medicine of The University of South Dakota, 414 East Clark Street, Vermillion, SD, USA

## Abstract

**Background:**

Mifepristone (MF) has been largely used in reproductive medicine due to its capacity to modulate the progesterone receptor (PR). The study of MF has been expanded to the field of oncology; yet it remains unclear whether the expression of PR is required for MF to act as an anti-cancer agent. Our laboratory has shown that MF is a potent inhibitor of ovarian cancer cell growth. In this study we questioned whether the growth inhibitory properties of MF observed in ovarian cancer cells would translate to other cancers of reproductive and non-reproductive origin and, importantly, whether its efficacy is related to the expression of cognate PR.

**Methods:**

Dose-response experiments were conducted with cancer cell lines of the nervous system, breast, prostate, ovary, and bone. Cultures were exposed to vehicle or increasing concentrations of MF for 72 h and analysed for cell number and cell cycle traverse, and hypodiploid DNA content characteristic of apoptotic cell death. For all cell lines, expression of steroid hormone receptors upon treatment with vehicle or cytostatic doses of MF for 24 h was studied by Western blot, whereas the activity of the G1/S regulatory protein Cdk2 in both treatment groups was monitored *in vitro *by the capacity of Cdk2 to phosphorylate histone H1.

**Results:**

MF growth inhibited all cancer cell lines regardless of tissue of origin and hormone responsiveness, and reduced the activity of Cdk2. Cancer cells in which MF induced G1 growth arrest were less susceptible to lethality in the presence of high concentrations of MF, when compared to cancer cells that did not accumulate in G1. While all cancer cell lines were growth inhibited by MF, only the breast cancer MCF-7 cells expressed cognate PR.

**Conclusions:**

Antiprogestin MF inhibits the growth of different cancer cell lines with a cytostatic effect at lower concentrations in association with a decline in the activity of the cell cycle regulatory protein Cdk2, and apoptotic lethality at higher doses in association with increased hypodiploid DNA content. Contrary to common opinion, growth inhibition of cancer cells by antiprogestin MF is not dependent upon expression of classical, nuclear PR.

## Background

While mifepristone (MF) was originally synthesized as an antiglucocorticoid agent, the realization of its affinity for the progesterone receptor (PR) expanded its study and application in the field of reproductive medicine for early termination of pregnancy, emergency contraception and menstrual cycle regulation [1,2]. More recently, MF emerged as a potential treatment of endocrine-related diseases such as uterine leiomyoma and endometriosis [3]. Moreover, the potential use of MF in oncology has been promising [4]. Because several tumors of both gynecologic and non-gynecologic origin are steroid hormone-dependent and express PR, MF has been investigated as a potential anti-cancer therapeutic agent largely based on its capacity to modulate PR. However, it remains unclear whether the mechanism through which MF acts to induce cytostasis and lethality in cancer cells actually requires PR expression.

Evidence suggests that the cytostatic effect of MF may be mediated by an agonistic action on PR. Support for this idea comes from studies using T47Dco breast cancer cells expressing high levels of PR, in which MF interfered with cell proliferation, displaying progesterone-like effects [5]. In MDA-MB-231 breast cancer cells that were transfected with PR, MF, akin to progesterone, inhibited cell growth by arresting cells in the G1 phase of the cell cycle [6]. Conversely, there is also evidence suggesting that the efficacy of MF as an anti-cancer agent may not require PR expression. Liang and colleagues reported that micromolar doses of MF alone were able to inhibit the growth of ER- and PR-negative MDA-MB-231 breast cancer cells [7]. Additionally, MF was capable of inhibiting the growth of LNCaP prostate cancer cells that were either androgen-sensitive or -refractory [8], while competition for PR and GR with equimolar doses of MF and progesterone or hydrocortisone could not reverse the degree of growth inhibition achieved by MF alone. Furthermore high concentrations of MF were unable to block growth inhibition induced by supra-pharmacological doses of progesterone (i.e. concentrations higher than needed to saturate the cognate PR) in endometrial cancer cells carrying PR [9]; instead high doses of MF potentiated the growth retardation and induction of apoptosis triggered by high doses of progesterone [10]. Such cytotoxicity of elevated concentrations of progesterone and MF was also observed in PR positive MCF-7 breast cancer cells and PR negative C4-I cervical carcinoma cells [11].These findings imply that MF may be working independently of either cognate PR or GR. On the other hand, competition for GR with an equimolar concentration of dexamethasone partially reversed growth inhibition by MF in the androgen-insensitive PC-3 prostate cancer cells, suggesting a possible role of GR in mediating the growth inhibitory properties of MF [12]. Altogether, findings from reports investigating the anticancer properties of MF as an endocrine-related phenomenon emphasize the lack of clarity regarding whether the mechanistic action of MF involves a specific steroid hormone receptor, although MF is mostly studied for anti-cancer therapy largely based on its anticipated interaction with PR.

In the few existing reports that investigated the effects of MF in epithelial ovarian cancer cells, our laboratory [13-15] and others [16,17], demonstrated the efficacy of MF as a growth inhibitory agent. We have shown that MF inhibits the growth of ovarian cancer cells of different genetic backgrounds in a dose-dependent manner *in vitro *and *in vivo *[13]. Our inquiry on the molecular mechanisms underlying MF-induced growth arrest revealed that ovarian cancer cells cultured in the presence of a cytostatic concentration of MF had reduced DNA synthesis and arrested the cell cycle at the G1/S phase transition [13]. Furthermore, exposure of OV2008 and SK-OV-3 ovarian cancer cells to a cytostatic concentration of MF increased the abundance of the cell cycle inhibitors p21^cip1 ^and p27^kip1^, decreased the abundance of Cdk2 and cyclin E, and decreased Cdk2 activity [13]. These results suggested that the cytostatic effect of MF is mediated through a G1 phase arrest [13]. However, when we exposed 6 different ovarian cancer cell lines of different genetic backgrounds and platinum sensitivities to concentrations of MF higher than 20 μM, the antiprogestin triggered cell death evidenced by an increase in sub-diploid fragmented DNA content and cleavage of caspase-3 and of its downstream substrate PARP [14]. Whether MF-mediated growth inhibition in ovarian cancer cells require PR remains unclear. The reported level of expression of PR in ovarian cancer cell lines is not without controversy. For example, Caov-3 cells were reported to express PR mRNA in one study [18] but not in another [19]. Similarly, studies in SK-OV-3 cells showing some or no expression of PR mRNA and protein have been published [19-23].

Up to this point, the literature has not addressed whether MF requires PR expression to work as an anti-cancer agent. In this study we sought to extend our findings in ovarian cancer cells to cancer cell lines of alternate tissues of origin, including cancers of both reproductive and non-reproductive origin, and questioned whether the expression of PR was related to the sensitivity of the cancer cells to the growth-inhibitory influence of MF. We hypothesized that MF would be capable of growth-inhibiting cancers of reproductive and non-reproductive origin regardless of PR expression, displaying cytostatic effects at lower micromolar concentrations and lethal effects at higher micromolar doses. We describe that MF inhibited the growth of 10 different cancer cell lines originating from the nervous system, breast, prostate, ovary, and bone, of which only one expressed cognate PR, suggesting that contrary to common opinion, the capability of MF to act as a growth inhibitory agent is unrelated to the expression of classical, nuclear PR.

## Methods

### Cell lines and in vitro exposure to MF

The human malignant meningioma IOMM-Lee cells were kindly provided by Dr. Anita Lal (University of California, San Francisco), the human malignant glioma U87MG cells, human osteosarcoma U-2OS and SAOS-2 cells, and estrogen-unresponsive breast carcinoma MDA-MB-231 cells were from the American Type Culture Collection (ATCC, Manassas, VA). These cell lines were maintained in Dulbecco's Modification of Eagle's Medium (DMEM) (Mediatech, Herndon, VA) supplemented with 10% fetal bovine serum (Atlanta Biologicals, Lawrenceville, GA), 10 mM HEPES (Mediatech), 4 mM L-glutamine (Mediatech), 1 mM sodium pyruvate (Mediatech), 100 IU penicillin (Mediatech) and 100 μg/ml streptomycin (Mediatech). SK-OV-3 and OVCAR-3 ovarian cancer cells were from ATCC, and routinely maintained in RPMI 1640 (Mediatech) supplemented with 10% fetal bovine serum (Atlanta Biologicals), 10 mM HEPES (Mediatech), 4 mM L-glutamine (Mediatech), 0.45% D-(+)-glucose (Sigma Chemical Co., St. Louis, MO), 1 mM sodium pyruvate (Mediatech), 1 X non-essential amino acids (Mediatech), 100 IU penicillin (Mediatech), 100 μg/ml streptomycin (Mediatech), and 0.01 mg/ml human insulin (Roche, Indianapolis, IN). The androgen-responsive human prostate carcinoma LNCaP and androgen-unresponsive PC-3 cells were from ATCC and maintained in RPMI 1640 (Mediatech) supplemented with 10% fetal bovine serum (Atlanta Biologicals), 10 mM HEPES (Mediatech), 4 mM L-glutamine (Mediatech), 1 mM sodium pyruvate (Mediatech), 100 IU penicillin (Mediatech), and 100 μg/ml streptomycin (Mediatech). The human breast adenocarcinoma MCF-7 cells were obtained from ATCC and maintained in DMEM (Mediatech) supplemented with 10% fetal bovine serum (Atlanta Biologicals), 10 mM HEPES (Mediatech), 4 mM L-glutamine (Mediatech), 1 mM sodium pyruvate (Mediatech), 100 IU penicillin (Mediatech), 100 μg/ml streptomycin (Mediatech), 1 X non-essential amino acids (Mediatech), and 0.01 mg/ml human insulin (Roche). All cell lines were cultured at 37°C in a humidified atmosphere in the presence of 5% CO_2_. Treatment of the cells with MF (Sigma) used a 20,000 μM stock solution of the drug in DMSO (Mediatech). The maximal concentration of DMSO in medium was 0.2% (v/v). We have shown previously that cancer cells responded similarly to micromolar concentrations of MF when cultured in media without phenol red and charcoal-extracted fetal bovine serum or media containing unextracted serum and having phenol red [13]. Consequently in this work all experiments were conducted using media with unextracted serum and in the presence of phenol red.

### Cell proliferation and doubling times

To measure proliferation in the presence of MF, cells were seeded into 6-well plates at a density selected to ensure exponential growth of each cell line while preventing the cells from reaching 100% confluence over the course of the experiment. Following a period of 24 h allotted for cell adherence, the cells were cultured in the continuous presence of MF or DMSO for 72 h. Triplicate cultures were trypsinized, pelleted by centrifugation at 500 *g *for 5 min, and resuspended in the appropriate growth medium. An aliquot of each cell suspension was combined with ViaCount reagent (Guava Technologies, Hayward, CA) resulting in a 1:10 (v/v) dilution and then studied using the Guava ViaCount application in the Guava EasyCyte Mini microcapillary cytometer (Guava Technologies) as we previously described [15]. The data were acquired and analyzed using the CytoSoft 4.1 software (Guava Technologies). When indicated, the concentrations of MF required to inhibit cell proliferation by 50% or IC_50 _were determined using software designed to study drug interaction that calculates the median effective dose, Dm, which is analogous to the IC_50 _(Calcusyn, Biosoft, Cambridge, UK). To obtain cell culture doubling times (DT), cells were plated at a density equal to that used in dose-response experiments and allowed to grow in culture for 96 h. Cells were harvested in triplicate and counted by microcytometry (Guava technologies) every 12 h. A nonlinear regression analysis designed to estimate the DT of exponentially growing cells was conducted for each growth curve using Prism 5.0 computer software (GraphPad, San Diego, CA) and an equation that fits a rate constant to a data set demonstrating exponential growth. The rate constant (K) was then used to calculate a value for DT as follows: DT = ln(2)/K.

### Cell cycle analysis

Cells exposed to either vehicle or MF for 72 h were trypsinized, pelleted by centrifugation at 500 *g *for 5 min, resuspended in the appropriate growth medium, and fixed with 4% paraformaldehyde. An aliquot ranging from 40,000-300,000 cells (varying by treatment and cell line) was then washed with PBS and pelleted by centrifugation at 500 *g *for 5 min. Cells were resuspended in 200 μl of cell cycle buffer [3.8 mM sodium citrate (Sigma), 7 U/ml RNase A (Sigma), 0.1% (v/v) Triton X-100 (Sigma), and 0.05 mg/ml propidium iodide (Sigma)] at a concentration of 200-1,500 cells/μl, and analyzed for the capacity of their DNA to bind propidium iodide utilizing the Guava EasyCyte Mini microcapillary cytometer and the cell cycle application of the CytoSoft 4.1 software.

### SDS-PAGE and Western blotting

Cells were scraped, pelleted, and washed twice with PBS, then snap frozen followed by storage at -80°C. Cells were lysed by the addition of NP-40 lysis buffer containing 50 mM Tris-HCl (pH 7.5), 150 mM NaCl, 0.5% NP-40 (Sigma), 50 mM sodium fluoride (Sigma), 1 mM PMSF (Sigma), 1 mM dithiothreitol (Invitrogen, Carlsbad, CA), 2 μg/ml pepstatin (Sigma), 2 μg/ml leupeptin (Sigma), 2 μg/ml aprotinin (Sigma), and 1 mM orthovanadate (Sigma). Lysates were centrifuged at 16,000 *g *for 20 min at 4°C, and the supernatant was considered the whole cell extract, which was assayed for protein content using the bicinchoninic acid method (BCA; Pierce, Rockford, IL). Whole cell extracts were appropriately diluted in 3 X concentrated electrophoresis sample buffer and boiled for 10 min. Equivalent amounts of protein (100 or 50 μg) were loaded in fixed 7.5% or 12% polyacrylamide gels, subjected to SDS-PAGE and transferred to PVDF membranes. The blots were blocked in 5% (v/v) nonfat milk in TBS containing 0.1% (v/v) Tween 20 (T), and then probed overnight with primary antibodies against PR (clone hPRa7; 4 μg/mL; Thermo Fisher Scientific, Fremont, CA; or #1483-1; 1:1,000; Epitomics, Burlingame, CA), GR (#sc-1003; 1:1,000; Santa Cruz Biotechnology, Santa Cruz, CA), AR (#1852-1; 1:10,000; Epitomics), ER-α (#4200-1; 1:1,000; Epitomics), p21^cip1 ^(clone 6B6; 2 μg/mL; BD Biosciences, San Diego, CA), p27^kip1 ^(clone 57; 1:2,000; BD Transduction Laboratories, San Diego, CA), cyclin E (clone HE12; 0.5 μg/ml; BD Pharmigen, San Diego, CA), and Cdk2 (M2; #sc-163; 1:1,000; Santa Cruz Biotechnology). The membranes were washed 3 × 5 min in TBS-T and incubated with a 1: 10,000 dilution of peroxidase-conjugate secondary antibody (#111-035-003 or #115-035-003; Jackson ImmunoResearch Laboratories, West Grove, PA) for 30 min at room temperature. The blots were again washed, developed by chemiluminescence, and exposed to radiographic film. Blots were also probed with an antibody directed against β-Actin (clone AC-15; 1:20,000; Sigma) to control for protein loading. When indicated, densitometry was used to compare protein expression using ImageJ 1.43 software (National Institutes of Health, Bethesda, MD).

### Cdk2 in vitro kinase assay

An aliquot (100 μg of protein) from each NP-40 cell lysate was incubated overnight at 4°C with constant rotation in 1 ml of NP-40 lysis buffer containing 1 μg polyclonal rabbit antibody to Cdk2 (M2; #sc-163; Santa Cruz Biotechnology). Immunocomplexes associated with Cdk2 were collected after incubating for 2 h with protein A/G PLUS-Agarose beads (Santa Cruz Biotechnology). The immune complexes were washed three times with NP-40 lysis buffer and twice with kinase buffer [50 mM HEPES (pH 7.2), 10 mM MgCl_2_, 1 mM DTT, 10 mM β-glycerophosphate, and 1 mM sodium fluoride]. Subsequently, the beads were resuspended in 30 μl of kinase buffer containing 2 μg histone H1 (Upstate Cell Signaling Solutions, Lake Placid, NY), 5 μM ATP (Upstate), and 5 μCi [γ^32^P]ATP (MP Biomedicals; Irvine, CA). The reaction mixtures were incubated at 30°C for 30 min and then terminated with 30 μl of 2 X electrophoresis sample buffer, boiled, and separated on 12% SDS-PAGE. Gels were stained with Coomassie blue (Sigma) to visualize the histone H1 bands, dried, and autoradiographed.

### Statistical analysis

All data are reported as mean ± SEM, and statistical significance was defined as *P <*0.05. To compare cell cycle kinetics and hypodiploid DNA distribution, one-way analysis of variance (ANOVA) followed by the Tukey's multiple comparison test was used as appropriate.

## Results

### MF inhibits the growth of tumor cells of the nervous system, breast, prostate, ovary, and bone

**Figure 1:**
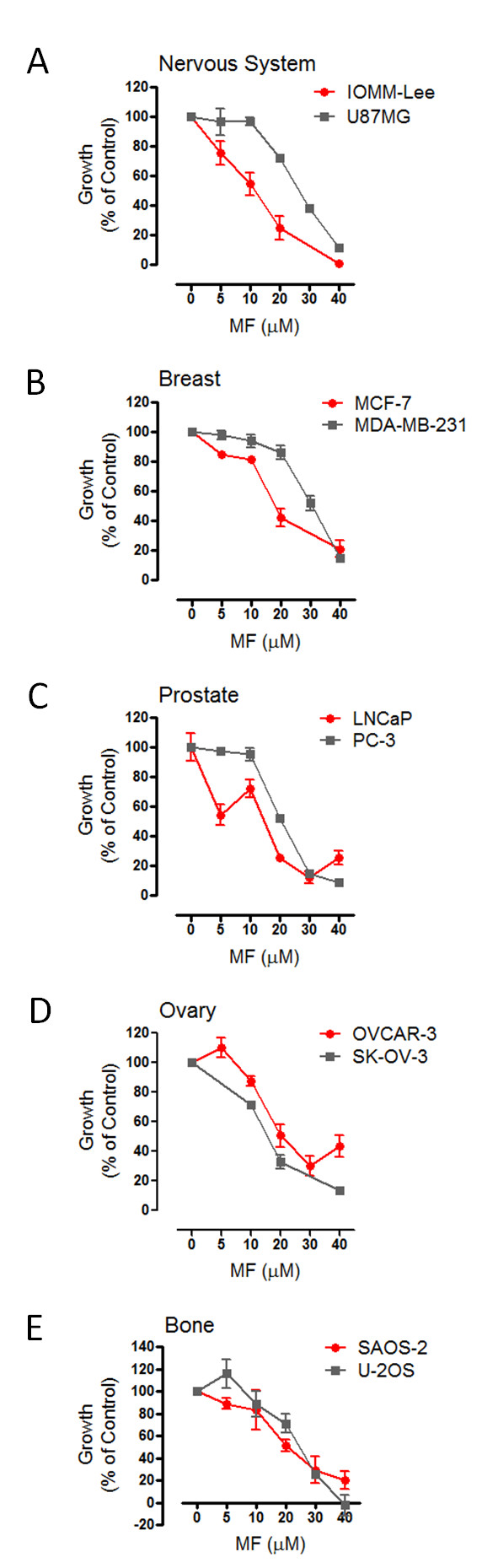
**MF inhibits the growth of tumor cell lines of the nervous system (A), breast (B), prostate (C), ovary (D), and bone (E)**. Cells were seeded at a density appropriate for each cell line, allowed to adhere for 24 h, and then exposed to the indicated concentrations of MF for 72 h. At the end of the experiment, cells were harvested by trypsinization and counted by microcytometry. Growth curves are expressed as the percent-growth of MF-treated cells with respect to vehicle-treated cell growth. The growth of vehicle-treated cells was calculated as the difference between the total number of cells at 0 h and 72 h of treatment, and was designated as 100% growth. Data points represent the mean ± s.e.m. of at least 3 independent experiments completed in triplicate.

A panel of 10 cancer cell lines was selected to assess the capacity of MF to inhibit the growth of cancer cells originating from the nervous system, breast, prostate, ovary and bone. A series of dose-response experiments was conducted to study the effect of 72 h exposure to MF on cell growth. In accordance with our hypothesis, MF inhibited the growth of the entire panel of cancer cells included in the study in a dose-related manner (Figure 1). We compared the growth inhibitory potency of MF among cell lines quantitatively by comparing the mean IC_50 _values representing at least 3 independent experiments for each cell line (Table 1). The DT for each cell line was indicated in the table and its calculation depicted in Additional File 1, Figure S1. Using this method, IOMM-Lee, LNCaP, and SK-OV-3 cells displayed lower IC_50s _to MF-induced growth inhibition while MCF-7, PC-3, OVCAR-3, U87MG, U-2OS, and SAOS-2 cells responded to MF at higher IC_50s_. MDA-MB-231 cells were the least sensitive to growth inhibition by MF as indicated by the highest IC_50_. Overall all cell lines responded to the growth inhibitory effect of MF with IC_50s _ranging from ~ 9 to 30 μM.

**Table 1:** Concentration of MF needed to achieve 50% growth inhibition (IC_50_) of the cell lines studied and their doubling times (DT)

Origin	Cell Line	IC_50 _(μM)	DT (h)
N. System	IOMM-Lee	9.4 ± 1.2	13.9
N. System	U87MG	23.2 ± 0.0	36.9
Breast	MCF-7	17.7 ± 2.0	37.7
Breast	MDA-MB-231	29.2 ± 1.3	33.3
Prostate	LNCaP	14.1 ± 0.5	50.7
Prostate	PC-3	18.5 ± 2.0	30.7
Ovary	OVCAR-3	19.0 ± 1.3	55.7
Ovary	SK-OV-3	12.6 ± 0.1*	36.9
Bone	U-2OS	21.9 ± 2.3	29.2
Bone	SAOS-2	18.7 ± 3.2	49.2

IC_50 _data are expressed as mean ± s.e.m of three experiments each one performed in triplicate. DT data are the result of one experiment performed in triplicate (see Additional file 1: Figure S1). *Date obtained from our previously published work [14]

### MF induces G1 accumulation in select cancer cell lines

**Figure 2:**
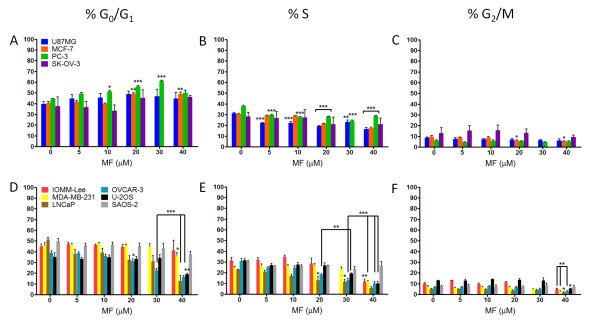
**Cell cycle traverse in cancer cell lines exposed to MF**. Cells were exposed to vehicle or the indicated concentrations of MF for 72 h, harvested, and stained with propidium iodide. DNA content was analyzed by microcytometry. Bars represent the mean ± s.e.m. of at least 3 independent experiments completed in triplicate, and show the percentage of cell particles with G0/G1 **(A, D)**, S **(B, E) **and G2/M **(C, F) **DNA content. *P < 0.05, **P < 0.01, ***P < 0.001 denote differences as compared to controls.

**Figure 3:**
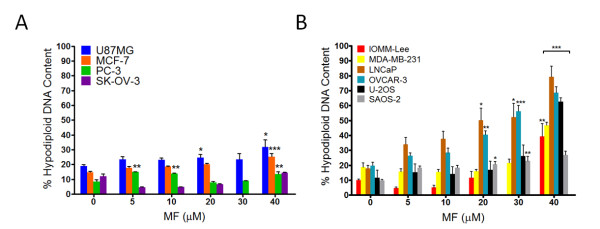
**Hypodiploid DNA content in MF-treated cancer cells**. Cells were exposed to vehicle or MF for 72 h, harvested, and stained with propidium iodide. DNA content was analyzed by microcytometry. Bars represent the mean ± s.e.m. of at least 3 independent experiments completed in triplicate, and show the percentage of cell particles with hypodiploid DNA content indicative of cell death. **(A) **A modest increase in the proportion of cells with hypodiploid DNA content was observed in cell lines that responded to MF by accumulating in G1 phase (see Figure 2). **(B) **A large, dose-dependent increase in the proportion of cell particles with hypodiploid DNA content was observed in cell lines that did not show an accumulation in G1 phase in response to increasing concentrations of MF (see Figure 2). *P < 0.05, **P < 0.01, ***P < 0.001 denote difference as compared to controls.

To analyze the effect of MF treatment on cell cycle traverse, each cell line was cultured in the presence of vehicle or MF for 72 h, harvested, and stained with propidium iodide; the distribution of cells with DNA content characteristic of G1, S, or G2/M phases of the cell cycle, as well as the proportion of cell particles with hypodiploid DNA content--encompassing particles from dead cells--was analyzed by microcytometry. Figure 2 represents the cell cycle distributions of all cell lines following exposure to MF. Consistent with our previous findings in ovarian cancer cells, we observed an accumulation of cells with DNA content representative of G1 phase in the U87MG, MCF-7, PC-3, and SK-OV-3 lines (Figure 2A). A significant accumulation of cells in G1 phase was observed at a concentration of 10 μM of MF in PC-3 cells and of 20 μM of MF in MCF-7 cells. While statistical significance was not achieved with U87MG and SK-OV-3 cells, the trend of G1 accumulation was apparent. G1 accumulation occurred in a dose-dependent manner in these 4 cell lines, with a slight decrease at a concentration of 40 μM MF corresponding to an increase in hypodiploid DNA content, indicating cellular lethality at this high concentration of the steroid (see below; Figure 3). The accumulation of cells with G1 DNA content was accompanied by a dose-dependent decrease in the proportion of cells with DNA content characteristic of S and G2/M phases (Figure 2B and 2C). Alternately, cell cycle distributions of IOMM-Lee, MDA-MB-231, LNCaP, OVCAR-3, U-2OS and SAOS-2 lines did not show an apparent accumulation of cells with G1 DNA content upon 72 h exposure to MF (Figure 2D). Instead, the proportion of G1 DNA content remained fairly constant at lower doses of MF and then decreased as these cell lines became susceptible to the lethality of higher drug concentrations. In this subset of cell lines, we also observed a decrease in the proportion of cells with DNA content characteristic of S and G2/M phases that was most notable at higher concentrations of MF (Figure 2E and 2F).

### Cancer cell lines associated with MF-induced G1 arrest exhibit lower lethality at a high concentration of MF

Cell particles with hypodiploid DNA content were considered to represent the proportion of dying cells. We observed that the subset of cell lines that did display an accumulation in G1 phase upon exposure to MF appeared less susceptible to lethality induced by high concentration of MF than those that did not (Figure 3A and 3B). The mean difference in the proportion of cells with hypodiploid DNA content between 0 h and 72 h of treatment was calculated for each cell line, and then the mean lethality induced by the highest concentration of MF (40 μM) was calculated for both subsets of cell lines. Cells accumulating in G1 phase displayed a modest induction of lethality by 40 μM MF ranging from 5.3 ± 2.7% (PC-3) to 12.9 ± 5.1% (U87MG), with a mean of 8.7 ± 1.8% (Figure 3A). Cell lines without G1 accumulation were more sensitive to MF-induced lethality, ranging from 17.0 ± 2.1% (SAOS-2) to 61.3 ± 7.3% (LNCaP) with a mean lethality of 39.3 ± 6.9% (Figure 3B). In summary, when we compared the difference between the mean proportion of cells with hypodiploid DNA content at 0 h of treatment and after 72 h of exposure to 40 μM of MF in all cancer cell lines, we found that a lack of observable G1 accumulation in response to MF was associated with significantly greater lethality.

### The cytostatic effect of MF in the cancer cells studied is associated with a decline in the activity of Cdk2

**Figure 4:**
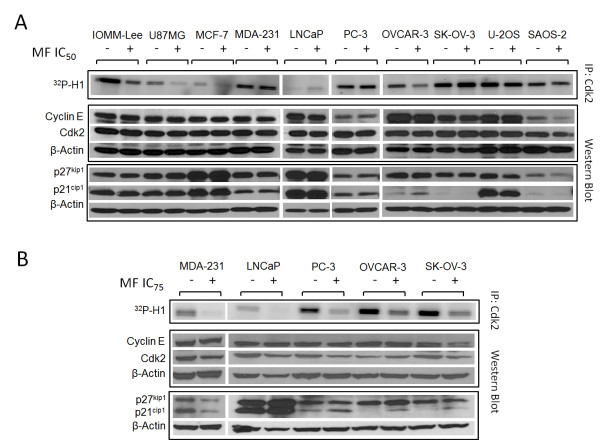
**Activity of Cdk2 and abundance of G1/S cell cycle regulatory proteins in MF-treated cancer cells**. Whole-protein extracts from cells treated with either vehicle (-), the IC_50 _(**A) **or the IC_75 _**(B) **concentrations of MF (+) for 24 h. The whole cell extracts were either immunoprecipitated with anti-Cdk2 antibody and assayed for their capacity to phosphorylate histone H1 *in vitro *in the presence of [^32^P]ATP (upper panels in A and B), or separated by electrophoresis and the immunoblots probed with antibodies against the cell cycle regulatory proteins p21^cip1^, p27^kip1^, cyclin E, and Cdk2. β-actin was included as a protein loading control (lower panels in A and B). Cyclin E and Cdk2 were immunoblotted in one membrane whereas p21^cip1 ^and p27^kip1 ^were blotted in a separate membrane. Consequently, each membrane was blotted separately with anti-β-actin. This experiment was repeated twice with a similar outcome. MDA-231 means MDA-MB-231.

Cdk2, in association with cyclin E, is mostly responsible for allowing cells to engage the process of DNA synthesis during the S phase of the cell cycle, whereas p21^cip1 ^and p27^kip1 ^are usually involved in inhibiting cyclin E/Cdk2 activity [24]. Previous work in our laboratory has pointed to a reduction in Cdk2 activity as a potential mechanism of MF-induced growth arrest [13]. We therefore questioned whether a similar reduction in the activity of this enzyme would be translated to this panel of cell lines expanding various cancers. In all cells lines exposed to either MF IC_50 _and/or IC_75_, the activity of Cdk2 was reduced when treated with MF (Figure 4A and 4B). The decline in Cdk2 activity induced by MF was accompanied by increased p21^cip1 ^abundance in U87MG, MCF-7, OVCAR-3, LNCaP, PC-3 and SK-OV-3 cells; increased p27^kip1 ^levels in U87MG, MCF-7, LNCaP, and OVCAR-3 cells; decreased levels of Cdk2 in SAOS-2, MDA-MB-231, LNCaP, and PC-3 cells; and reduced cyclin E in SAOS-2 and SK-OV-3 cells.

### The efficacy of MF as a cytostatic agent in cancer cells is not related to the expression of progesterone receptor, androgen receptor, or estrogen receptor

**Figure 5:**
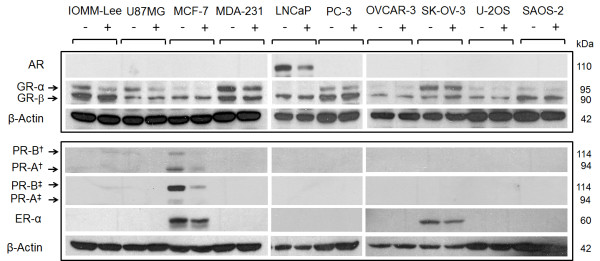
**Expression of progesterone, androgen, glucocorticoid and estrogen receptors in cancer cells exposed to MF**. Cells were exposed to either vehicle or the IC_50 _concentration of MF specific to each cell line for 24 h. Cells were subsequently harvested, lysed, and whole-protein extracts (50 μg for GR and AR; 100 μg for PR and ER-α) were separated by electrophoresis. Immunoblots were then probed with the indicated antibodies (for PR: †clone hPRa7, Thermo Fisher Scientific; ‡ #1483-1, Epitomics); two commercially available antibodies for human PR were used to strengthen the reliability of the results. β-actin was included as a control for protein loading. Because AR, GR-α and GR-β were immunoblotted in one membrane and PR-A, PR-B and ER-α were blotted in a separate membrane, each membrane was blotted separately with anti-β-actin. This experiment was repeated twice with similar outcome. MDA-231 means MDA-MB-231.

Since MF was effective in growth inhibiting the 10 human cancer cell lines included in this study, we subsequently evaluated in all cells the expression levels of classical, nuclear PR isoforms A (PR-A) and B (PR-B), GR isoforms alpha (GR-α) and beta (GR-β), AR, and ER isoform alpha (ER-α) to determine whether there is a correlation between the expression of one or more receptor types and the sensitivity to MF-induced growth inhibition when MF is used at cytostatic, non-lethal doses. We were particularly interested in the role of classical PR isoforms which are anticipated to be the primary targets and mediators of the antiprogestin activity of MF [25]. We also decided to study the expression of receptors in the absence or presence of MF considering that previous reports suggest that GR and PR are modulated by the drug [17,26]. Cells were grown to 50-70% confluence and were exposed to either vehicle or an IC_50 _concentration of MF for 24 h prior to harvesting. Cells were lysed, and whole protein extracts were immunoblotted for the presence of PR-A, PR-B, GR-α, GR-β, AR, and ER-α. Figure 5 shows the steroid hormone receptor expression levels in all cell lines treated with either vehicle or MF. In vehicle-treated cells, utilizing two different anti-human PR primary antibodies that recognize both PR isoforms with varied efficacy, we detected PR-A and PR-B only in MCF-7 breast cancer cells. MCF-7 and SK-OV-3 cells expressed ER-α, whereas only prostate LNCaP cells expressed AR. All cell lines expressed GR-β, while GR-α appeared to be nearly absent or at very low concentrations in MCF-7, LNCaP, OVCAR-3, U-2OS, and SAOS-2 cells, but more abundant in IOMM-Lee, U87MG, MDA-MD-231, PC-3, and SK-OV-3 cells. Additionally, in response to a cytostatic concentration of MF, we found a decrease in the abundance of nearly all steroid hormone receptors expressed. Both PR isoforms decreased in MCF-7 cells following 24 h exposure to MF regardless of the PR antibody utilized. Similarly, the expression of AR in LNCaP prostate cancer cells exposed to MF decreased, a slight decrease in the abundance of ER-α was observed in MCF-7 and SK-OV-3 cells, whereas GR-α slightly declined in some but not all cell lines treated with MF. Conversely GR-β did not apparently change among vehicle-treated vs. MF-treated cells in any investigated cell line.

## Discussion

We have shown that MF is able to inhibit the growth of cancer cells derived from the nervous system, breast, prostate, ovary, and bone, with nearly all of them not expressing the classical, nuclear PR. Mainstream literature on the anti-cancer effect of MF assumes that it acts as a PR antagonist, which implies that PR in the target tissue is a pre-requisite for MF's anti-growth activity. Our present work challenges such a dogma, opening the field of study to alternate, non-classical mechanisms whereby MF operates as a cell growth inhibitor without the necessity of nuclear PR being present or operational. If these results were translated into the clinic, the presence or absence of classical, nuclear PR would not be relevant and would not impact the usage of this drug for cancer therapy.

Dose-response experiments, in which a panel of 10 cancer cell lines were exposed to increasing concentrations of MF, indicated that micromolar doses of MF were effective to inhibit the growth of malignant meningioma IOMM-Lee cells, glioblastoma U87MG cells, breast cancer MCF-7 and MDA-MB-231 cells, prostate cancer LNCaP and PC-3 cells, ovarian cancer OVCAR-3 and SK-OV-3 cells, and osteosarcoma U-2OS and SAOS-2 cells; in all cell lines studied MF was cytostatic at lower micromolar doses but lethal at higher micromolar concentrations. We selected the U87MG cell line based on the aggressive nature of malignant glioma, their reported lack of PR expression [27], and the reported efficacy of MF in delaying their growth both *in vitro *and *in vivo *[27]. The malignant meningioma IOMM-Lee cell line [28] was the second line selected from the nervous system because over 70% of meningiomas express PR [29], and clinical trials conducting long-term MF treatment of patients with unresectable meningioma have been promising [30,31]; yet the expression of PR in IOMM-Lee was, to our knowledge, unknown. Our study shows that MF growth arrests IOMM-Lee malignant meningioma cells which lacked PR expression, suggesting that the presence of PR may not be required for MF to operate as a growth inhibitor in this cancer type. MCF-7 breast cancer cells are known to be responsive to estrogen and to express PR and ER [32-34], while triple negative MDA-MB-231 (i.e., cells lacking PR, ER-α, and HER2) are highly aggressive [7]. Previous studies demonstrated the efficacy of MF as single agent or in combination with 4-hydroxy-tamoxifen in growth-inhibiting both cell lines [7,35-38]. Our data confirm those results and find that triple negative MDA-MB-231 cells are slightly less responsive to MF than estrogen-responsive MCF-7 cells (Table 1). Similarly in LNCaP and PC-3 prostate cancer cells, classified respectively as androgen-sensitive and androgen-insensitive, MF was efficacious in growth-inhibiting both cell lines, confirming previous data using androgen-refractory LNCaP cell variants *in vitro *[8,12] and *in vivo *[12,39]. PR does not appear to be related to primary prostate tumors, but increased PR expression was observed in prostate metastasis [40]. LNCaP cells were reported to express PR-A and PR-B mRNA, yet PC-3 were shown not to express PR [41]. We were unable to detect PR proteins in either of these cell lines but found that LNCaP cells were slightly more responsive than PC-3 cells to MF (Table 1), which may be related to the capacity of MF to partially bind AR that are present in LNCaP but not in PC-3 [42]. In ovarian cancer, OVCAR-3 cells have been reported to express PR only *in vivo *upon estradiol stimulation [19], while as indicated earlier in the introduction, PR expression in SK-OV-3 ovarian cancer cells is controversial. Both cell lines were shown to be sensitive to growth inhibition by MF *in vitro *[13,16] and *in vivo *[13], what we further confirm in this study. Once again, the presence of PR does not appear to be a pre-requisite for the cells to respond to MF. Finally, we studied two osteosarcoma cell lines of different genetic backgrounds, which were reported to express no PR and very low levels of GR-α [43]. We confirmed the lack of PR and the low levels of GR-α protein but instead found GR-β with values that were slightly higher in SAOS-2 than in U-2OS cells. While all cell lines studied were growth inhibited by MF, PR expression was observed only in MCF-7 breast cancer cells known to be estradiol-responsive and to express both PR-A and PR-B isoforms [32-34]. AR was observed only in the androgen-dependent LNCaP prostate cancer cells, which is consistent with the reported expression of AR when these cells were first characterized [44]. In accordance with the reported expression of ER in MCF-7 cells [45], we detected ER-α in this cell line. While only a few of the cell lines included in this study expressed PR, AR, and/or ER-α, all cell lines were sensitive to the cytostatic and lethal effects of MF, suggesting that the expression of PR, AR, and ER-α is not required for MF to act as a growth inhibitor agent. Presence of MF reduced the expression of PR and ER-α in MCF-7 cells, AR in LNCaP cells, and ER-α in SK-OV-3 cells further discouraging the role of these receptors as mediators of the growth inhibitory effect of MF given that the cytostatic property of MF can be maintained long after those receptors are down-regulated.

Our previous studies in ovarian cancer cells indicated that MF-induced growth inhibition occurs through G1 cell cycle arrest and a profound inhibition of the G1/S kinase, Cdk2 [13]. In the present study, analysis of cell cycle kinetics following 72 h exposure of each cell line to MF showed the accumulation of cells with G1 phase DNA content in U87MG, MCF-7, PC-3, and SK-OV-3 cells, yet 6 cell lines in this study did not respond to MF with G1 arrest. Instead, we observed either a maintenance of the proportion of cells in each phase of the cell cycle up to a lethal concentration of MF (IOMM-Lee, MDA-MB-231, U-2OS, and SAOS-2 cells), or a steady decline in the proportion of cells in each phase of the cell cycle beginning at low concentrations of MF with a corresponding increase in cells with hypodiploid DNA content (LNCaP and OVCAR-3 cells). Despite these differences, the decline in the activity of Cdk2 within 24 h of exposure to MF was a commonality among the 10 cell lines studied (Figure 4), as we have shown in ovarian cancer cells for MF [13] and more recently for two other antiprogestins, ORG-31710 and CDB-2914 [46].

In this work evidence has ruled out that classical, nuclear PR must be expressed in a cancer cell to respond to the cytostatic activity of the so-classified antiprogestin MF. Further studies will need to be conducted to define the molecular targets and mediators of MF's action. Though all cell lines studied express variable levels of GR-α and GR-β, we could not find any correlation between the relative growth inhibitory response of the cells to MF and the relative abundance of GR-α (r = - 0.1831; *P *= 0.612), GR-β ( r = 0.0834; *P *= 0.818) or the ratio GR-α/GR-β (r = - 0.2366; *P *= 0.510) as determined by densitometry analysis of the Western blots presented in Figure 5 and corrected by β-actin loading. MF was designed in the mid-1980s with the purpose of treating Cushing's syndrome by working as a potent anti-glucorticoid agent [4,47]. Indeed MF binds GR-α with mostly antagonistic activity; yet it may have agonistic potency depending on the concentration of GR in the cell [48]. Although GR-β has been considered a dominant-negative regulator of GR-α [49-51], it was also reported that MF was the only compound of 57 potential natural and synthetic ligands to bind to the GR-β receptor isoform, and that interaction of GR-β with MF led to its nuclear translocation [52]. Additionally, this latter study found that despite its classification as a dominant-negative isoform lacking transcriptional activity, GR-β was capable of regulating gene expression in the absence of GR-α, and this activity was modulated by interaction with MF. A more recent study also reported intrinsic transcriptional activity of GR-β independent of GR-α, but neither found an association between MF binding and nuclear translocation of GR-β nor could detect modulation of GR-β transcriptional activity by MF [53], adding controversy to the actual activity of MF on GR-β. This evidence and our results strongly suggest that further studies need to be conducted to determine any role of either GR isoform on the anti-growth activity of MF.

A possibility exists in that the newly discovered progesterone receptor membrane component 1 (PGRMC1) [54,55] or the family of membrane PRs (mPRα, β, γ, δ, ε) [55-57] mediate the anti-tumor effects of MF. For instance, PGRMC1 expression increases while cognate, nuclear PR decreases in advanced stages of ovarian cancer, and overexpression of PGRMC1 interferes with the lethality of cisplatin, suggesting a survival role for PGRMC1 in ovarian cancer development [58]. In a panel of ovarian cancer cell lines expressing abundant mPRα, mPRβ, and mPRγ, but not classical nuclear PR-A and -B, exposure to progesterone mediated the expression of pro-apoptotic proteins via activation of JNK and p38 MAPKs [22]. Given that at micromolar concentrations MF functions as an agonist on both mPRα and mPRγ when expressed in yeast [59], it is conceivable that MF may mediate antiproliferation of cancer cells acting as an agonist of mPRs.

At micromolar doses, MF may have an alternate mechanism of action. For instance MF has a potent antioxidant effect when used at micromolar concentrations and attributed to the presence of the dimethylamino phenyl side chain of the molecule [60]. In endometrial cells and macrophages, the growth inhibitory effect of MF was partially attributed to the antioxidant property of the compound [61,62]. A putative antioxidant property of MF in cancer cells would be relevant in the context of p21^cip1 ^induced G1 arrest as p21^cip1 ^has been shown to be induced by some antioxidants in a p53-independent manner [63,64]. Another potential mechanism involved in MF's anti-growth activity is the induction of stress of the endoplasmic reticulum. For instance a recent study showed that MF induced an atypical unfolded protein response (UPR) in non-small lung cell carcinoma cells [65].

## Conclusions

This study has shown that the canonical, nuclear PR is not required for MF to successfully inhibit the growth of a panel of 10 cancer cell lines of different genetic backgrounds, hormone-responsiveness, and tissues of origin. Our study is limited only to eliminate the dogma that classical PR should be present to consider the use of MF in cancer therapy. However, the present results warrant mechanistic studies to uncover the ultimate mediators of MF's anti-proliferative activity in cancer cells. The role of mPRs, PGRMC1 and GR-β as mediators of MF anti-growth activity needs investigation. Furthermore the role of the endoplasmic reticulum responding to MF triggering the UPR, what could lead to either survival with cytostasis or death depending upon the concentration of MF, is a provoking hypothesis that deserves to be investigated.

## Competing interests

The authors declare that there is no conflict of interest that could influence the impartiality of the research reported.

## Authors' contributions

CRT carried out most experiments and drafted the manuscript. AAG performed the Cdk2 *in vitro *kinase assays and assisted CRT in some of the experiments. BNB performed the cell cycle analysis of the osteosarcoma cell lines and assisted CRT with the art work. CTO performed the experiments to calculate the doubling times of the cell lines studied. CMT conceived the study and contributed to the writing of the final version of the manuscript. All authors analyzed the results, read and approved the final manuscript.

## Pre-publication history

The pre-publication history for this paper can be accessed here:

http://www.biomedcentral.com/1471-2407/11/207/prepub

## Supplementary Material

Additional File 1 **Figure 1S. Growth curves displaying proliferation of vehicle-treated cancer cells as a function of time in culture**. Cells were seeded at a density equivalent to that used in dose-response experiments, and were allowed to grow in culture for 96 h. Triplicate wells were harvested by trypsinization and counted by microcytometry every 12 h. Data points represent the mean ± s.e.m. of one experiment completed in triplicate. A nonlinear regression designed to calculate the doubling time (DT) of exponentially growing cells was conducted to determine the proliferation rate of each cell line. Growth curves (●) and linear regression curves (▬) were generated using Graphpad Prism 5 software. Click here for file
