# Supplementary figures and images for: Antiprogestin mifepristone inhibits the growth of cancer cells of reproductive and non-reproductive origin regardless of progesterone receptor expression
Source: BMC Cancer. 2011 May 27;11:207. doi: 10.1186/1471-2407-11-207 (PMC3125282; doi:10.1186/1471-2407-11-207)

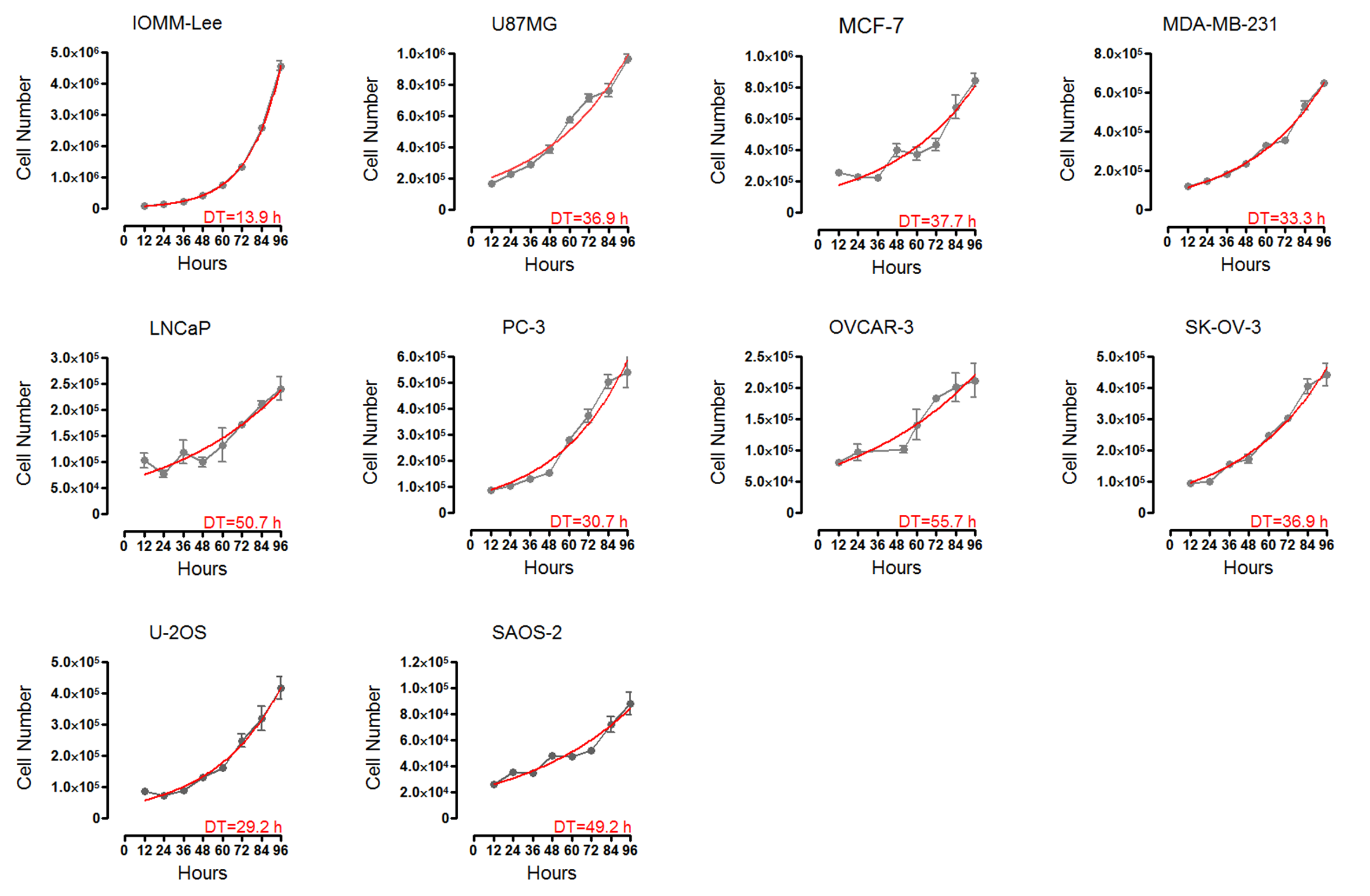

Supplement: Additional File 1 — Figure 1S. Growth curves displaying proliferation of vehicle-treated cancer cells as a function of time in culture. Cells were seeded at a density equivalent to that used in dose-response experiments, and were allowed to grow in culture for 96 h. Triplicate wells were harvested by trypsinization and counted by microcytometry every 12 h. Data points represent the mean ± s.e.m. of one experiment completed in triplicate. A nonlinear regression designed to calculate the doubling time (DT) of exponentially growing cells was conducted to determine the proliferation rate of each cell line. Growth curves (●) and linear regression curves (▬) were generated using Graphpad Prism 5 software. [file 1471-2407-11-207-S1.TIFF]
